# Ovarian Transcriptomic Analysis Reveals Differential Expression Genes Associated with Cell Death Process after Selection for Ovulation Rate in Rabbits

**DOI:** 10.3390/ani10101924

**Published:** 2020-10-20

**Authors:** Marta Serna-García, Rosa Peiró, Eva Serna, María Antonia Santacreu

**Affiliations:** 1 Instituto de Ciencia y Tecnología Animal, Universitat Politècnica de València, 46022 Valencia, Spain; garserma@hotmail.com; 2 Faculty of Agricultural and Veterinary Sciences, São Paulo State University, FCAV/UNESP, Jaboticabal 14884-900, São Paulo, Brazil; 3 Instituto de Conservación y Mejora de la Agrodiversidad Valenciana, Universitat Politècnica de València, 46022 Valencia, Spain; ropeibar@btc.upv.es; 4 Department of Physiology, Faculty of Medicine, University of Valencia, 46010 Valencia, Spain

**Keywords:** ovulation rate, litter size, transcriptomic analysis, rabbit, ovarian tissue

## Abstract

**Simple Summary:**

Transcriptomic analysis showed nineteen potential biomarkers in ovarian tissue from females belonged to a rabbit line selected for ovulation rate for 10 generations and the control line. These females differed not only in ovulation rate but also in prenatal survival since similar litter size were observed.

**Abstract:**

Litter size is an essential trait in rabbit meat production but with low heritability. A selection experiment for ovulation rate has been performed for 10 generations to improve litter size in rabbits. The selected line increased two ova more than the control line but nevertheless a negative correlation was observed with prenatal survival. A transcriptomic study was performed, using microarrays, in ovarian tissue from females belonging to the selected line and the control line. Our results showed 1357 differential expressed genes and nineteen potential biomarkers associated with prenatal mortality, which could explain differences between litter size in rabbits. Cell death was the most relevant process.

## 1. Introduction

Ovulation rate and prenatal survival are the main components of litter size in rabbits and pigs. Selection by ovulation rate was proposed, expecting a more efficient improvement in litter size than direct selection. Ovulation rate has been increased with success in the only selection experiment by ovulation rate developed in rabbits. After 10 generations of selection, the selected line (OR line) showed around two ova more than the control line (C line). However, litter size was not improved mainly due to a decrease of 12% in prenatal survival [1,2,3]. Similar results were found in pigs after selection by ovulation rate [4,5,6,7]. Several authors have proposed that a part of the reduction of prenatal survival in females with high ovulation rate could be due to a lower oocyte quality [8,9]. Follicular size, follicular maturity, and specific hormone and protein concentrations in the follicular fluid are related to the oocyte quality, which is required to restart meiosis, fertilization, and early embryo development [10]. Moreover, corpora lutea after ovulation play an important role in the production of progesterone. It is widely known that progesterone is essential to achieve and maintain pregnancy and the timing of the post-ovulatory progesterone rise is critical to the embryonic development and survival.

Genome-wide expression profiles could be a fundamental strategy to study changes in gene expression after selection based on a trait and to understand the biological processes involved in the expression of selected and related traits. The aim of this paper is to analyze, using microarrays, the transcriptomic profile of ovary tissue from females belonging to the OR line selected by ovulation rate and its control line. Our study can contribute to the understanding of the transcriptome events in ovarian tissue after selection by ovulation rate and offer a theoretical basis to further improve oocyte quality and, therefore, embryo survival and litter size.

## 2. Materials and Methods

### 2.1. Ethics Statement

All experimental procedures were approved by the Universitat Politècnica de València Research Ethics Committee, according to Council Directives 98/58/EC and 2010/63/EU.

### 2.2. Animals and Experimental Design

A total of six nulliparous rabbit females were used to perform the analyses. Three females belonging to the line selected for 10 generations for ovulation rate (OR line), and the other three females belong to the C line [3]. These three control females were the offspring of a cryopreserved population from generation zero of a OR selection experiment to avoid the effect of the cryopreserved technique.

Females were housed at the farm of the Universitat Politècnica de València in individual cages (flat-deck) with a extractable nest box with isolated plastic, and they were reared under a photoperiod of 16-h light: 8-h dark.

Ovulation is usually induced by coitus in the rabbit. Natural mating was carried out at 20 weeks old, and females were slaughtered by stunning and exsanguination 16 h later. After slaughter, the complete reproductive tract was removed. Ovary tissue (30–35 mg) from each female were collected in a tube with 200 μL of RNAlater (Ambion), frozen at −196 °C in liquid nitrogen, and then stored at −80 °C until RNA extraction.

### 2.3. Total RNA Extraction and Transcriptome Analysis

Total RNA was isolated directly using Trizol reagent following the manufacturer’s instructions (TRIzol, Invitrogen, Thermo-Fisher Scientific). RNA integrity number (RIN) was tested by the 2100 Bioanalyzer (Agilent Technologies, Santa Clara, CA, USA), and RNA concentration and purity were determined using a spectrophotometer (GeneQuant, GE Healthcare, Amersham Biosciences, Missouri, TX, USA). The purity and integrity of total RNA were similar and then comparable (*p*-values = 0.8).

GeneChip^®^ Rabbit Gene 1.0 ST Array allows us to analyze 23,282 well-annotated genes with 496,321 different probes with a median of 22 probes per gene. The databases used to design the arrays were Orycun 2.0 and Ensemble (May 2009). Raw data has been deposited in ArrayExpress, a publicly accessible database, with accession number E-MTAB-9406.

CEL files were imported into Partek Genomics Suite v6.6 (Partek, Inc., St. Louis, MO, USA) as CEL files. Raw data were pre-processed, including background correction, normalization, and summarization using robust multi-array average (RMA) analysis and then log2-transformed using Expression Console™ 1.4.1.46 software from Affymetrix.

One-way ANOVA analysis was performed with Partek Genomics Suite 6.6 software (Partek Inc., St. Louis, MO, USA) to identify differentially expressed genes. A *p*-value lower than 0.05 was considered as statistically significant. Principal component analysis (PCA) was performed to determine the significant sources of variability in the datasets. PCA reduces the complexity of high-dimensional data and simplifies, identifying expression patterns and sources of variability in a large dataset in a tridimensional fashion. The distance between any pair of points is related to the similarity between two samples in high-dimensional space, in this case, each variable corresponding to a one-dimensional space. Later, the genes were ordered according to their expression levels in unsupervised hierarchical clustering. Finally, the differentially expressed genes were imported into Pathway Studio version 10 (Elsevier Inc., Rockville, MD, USA) to identify the main biological processes and pathways with database ResNet v11. An enrichment *p*-value lower than 0.05 was considered as statistically significant.

## 3. Results

### 3.1. Transcriptome Profile Analysis in Ovarian Tissue

**Figure 1:**
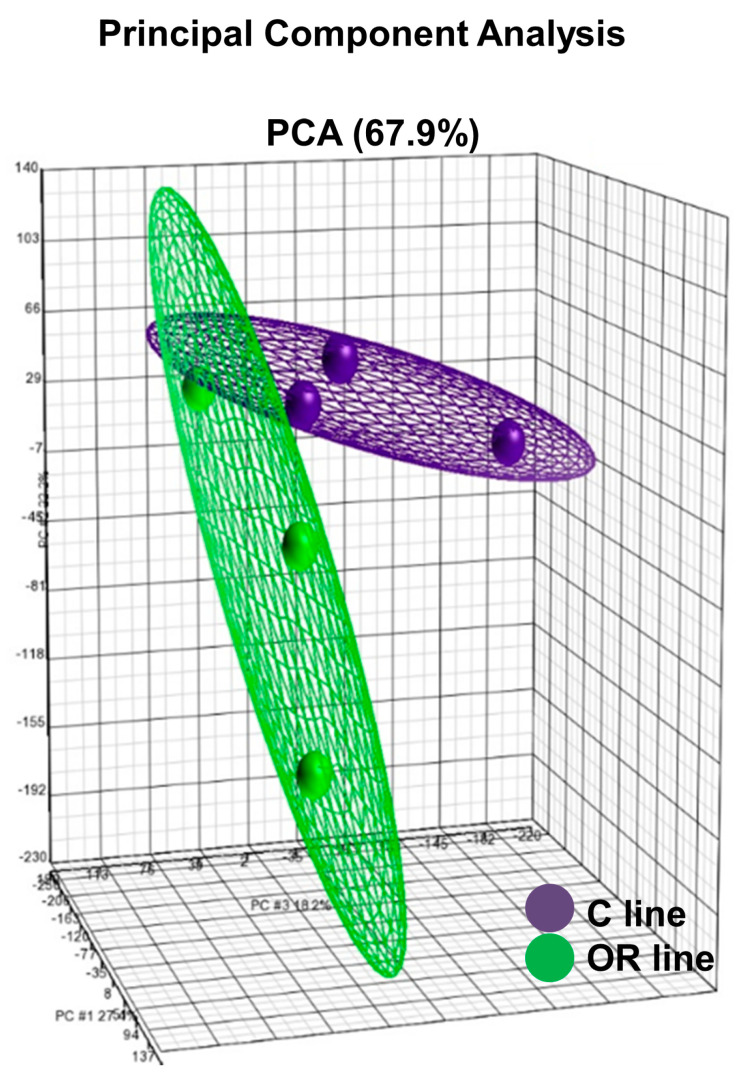
Principal component analysis (PCA) of the whole transcriptome of control line (C line, purple color) and line selected for ovulation rate during 10 generations (OR line, green color) in ovarian tissue. The total percentage of PCA mapping variability is 67.9%. Each data point represents one sample. The ellipsoids highlight the portioning of the different samples.

**Figure 2:**
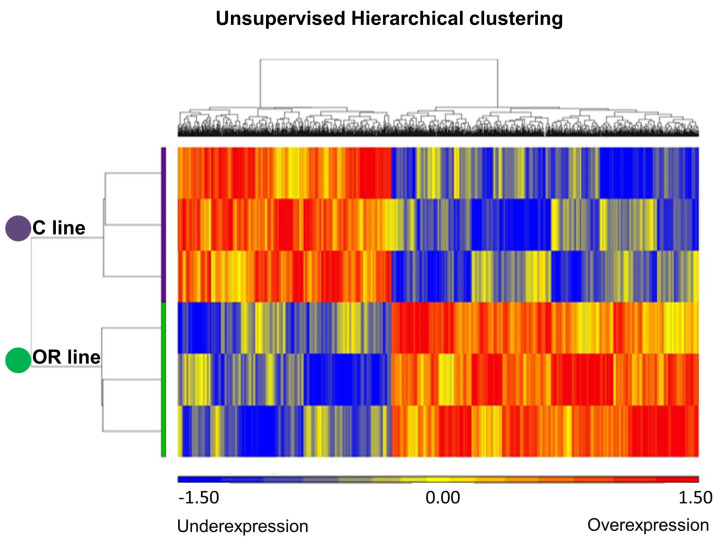
Unsupervised hierarchical clustering of 1357 differentially expressed genes in ovarian tissue. In purple, the control line (C line) clustered a completely different manner than the ovulation rate selected line (OR line, in green). Each line represents a sample and each column a gene. Overexpressed genes are represented in red and underexpressed genes in blue.

The principal component analysis showed the distribution of whole transcriptome in the six ovarian samples recovered 16 h after mating (Figure 1). Two clustered groups, corresponding to the control and the OR selected females, were observed and the percentage of variability of the three main components was 67.9%. Furthermore, a differential expression analysis was performed, and a total of 1357 differentially expressed genes (DEG) between females from the OR line vs. the control one was found (*p*-value < 0.05; see Table S1). Of these 1357 DEG, 922 were well-annotated which 529 genes were overexpressed and 393 were underexpressed in the OR line vs. the control line. Significant genes derived from the ANOVA analyses were visualized according to their expression levels in an unsupervised hierarchical clustering (Figure 2). These analyses confirmed that both groups, control and selected by OR, had different transcriptomic pattern.

### 3.2. Biological Analysis in Ovarian Tissue

To understand the biological contribution of the 1357 differentially regulated genes, Pathway Studio (Elsevier) was used. The most representative biological processes filter by enrichment *p*-value are represented in Table 1.

Moreover, cell process pathways most relevant in the differential gene expression analysis are showed in Table 2.

**Figure 3:**
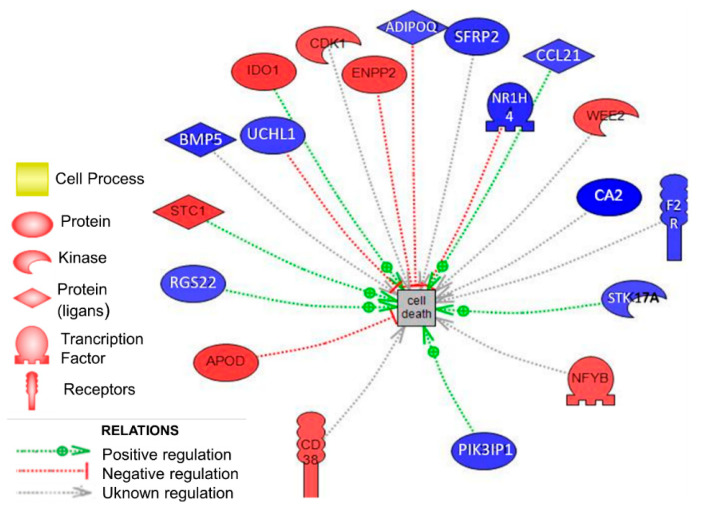
Customized subnetwork analysis focused on nineteen genes involved in cell death in ovarian tissue. Up-regulated (in red) and down-regulated (in blue) genes in the line selected for ovulation rate during 10 generations (OR line) vs. the control line (C line). The dashed line means the regulation relationship. Relations are colored by effect: Red color expressed a negative effect, green color expressed a positive effect, and grey expressed unknown effects.

We highlighted the apoptosis process because in both Table 1 and Table 2, it is a relevant process. For this reason, a custom subnetwork analysis focused on DEG involved in cell death in ovarian tissue was performed (Figure 3). Nineteen genes directly related to this biological process were found; eight overexpressed and 11 underexpressed in the OR line vs. the control line filtered *p*-value < 0.05 and fold change of│1.5│. These nineteen genes represented almost 60% of the total DEG well-annotated (Table 3).

## 4. Discussion

Litter size is one of the characters with the greatest economic importance in rabbit production [11], and, therefore, maternal lines are selected mainly by litter size [12]. The selection by ovulation rate has been proposed as a way to improve litter size more successfully. In only one selection experiment on ovulation rate performed in rabbits, an increase of approximately two ova were obtained; however, there was no success in improving litter size. This failure to increase litter size despite the increase in the ovulation rate was due to a decrease in prenatal survival (almost 12% per generation). The increase in prenatal mortality occurred both in the embryonic and fetal periods [1,2,3]. Laborda and co-workers suggested that a part of the increase in prenatal mortality found in the experiment of selection for ovulation rate could be due to the reduction in oocyte quality since females with high ovulation rate from selected line showed a lower concentration of glutathione [13]. Glutathione is considered as a relevant biochemical marker of the cytoplasmic maturation and consequently of the development and viability of oocytes [14]. In this study, some possible causes of this decrease in prenatal survival were described in terms of transcriptomic results. Microarray-based expression profiling allows characterizing more broadly, causing a specific phenomenon such as a decrease in prenatal survival resulting in no changes in litter size when all the genes are interrogated in a single experiment. It also helps to understand the main biological processes involved in this event.

We found 1357 DEG between the OR line and the control line in ovarian tissue. Most relevant gene expression changes were related to apoptosis and cell death. Apoptosis, a programmed cells death, plays a role on all the stages of oogenesis and even after ovulation [15]. There are several players responsible for oocyte apoptosis such as premature disruption of gap junctions, signal molecules, meiotic competency, and apoptotic factors. In the mentioned review is stated that the generation of reactive oxygen species in oocytes after ovulation regulates anti-apoptotic, proapoptotic, and apoptotic factors. We highlight that one of our biomarkers, Stanniocalcin 1 (STC1) gene was detected in rabbit ovarian tissue for the first time. Our study shows that the overexpression of STC1 could exert a positive regulation in programmed cell death in OR line. STC1 gene encodes a glycoprotein involved in many biological processes such as mineral homeostasis, angiogenesis, and steroidogenesis very relevant in ovarian function [16]. STC1 affects the progesterone production in granulosa cells, and progesterone is essential for embryo survival and development. For this reason, STC1 could have an essential function in corpora lutea. In humans, it is observed that protein A, regulated by STC1, is associated with pregnancy during follicle development [17].

Another up-regulated expression gene is Indoleamine 2.3-dioxygenase 1 (IDO)which causes T-cell apoptosis [18,19,20,21,22,23], anti-proliferative, and proapoptotic effects [24] like Apolipoprotein D (ApoD) [25]. The overexpression of glycoprotein ApoD in the OR line could be modulating immune function [19], stress response, lipid metabolism, ageing, and cell adhesion [26]. The importance of inflammatory response in the ovulatory process is highlighted by the commonalities and similarities between many of the events associated with ovulation and inflammation [27].

The participation of WEE1 homolog 2 (S. pombe) (protein kinase phosphorylates and inhibits family of cyclin-dependent kinase complexes (Cdks)) in cell cycle inhibiting the mitosis [28,29,30,31,32] and apoptosis [33,34,35,36] could be one of the reasons about for the failure in litter size in the OR line. This finding confirms that apoptosis is a relevant biological process corroborated in our results.

The presence of nuclear CD38 molecule during different hematopoietic differentiation stages suggests that it may play a role in the control of homeostasis [37] and regulate humoral immune responses [38]. This study shows an overexpression of this gene, and its deficiency could alter adipogenesis and lipogenesis [39], including neurotransmission, cell proliferation, apoptosis, bone remodeling, T lymphocyte signaling, and neutrophil migration [40]. This possible participation of an immune system down could be the consequence of finding chemokines downregulated like Chemokine (C-C motif) ligand 21 (CCL21). We also found that Phosphoinositide-3-kinase interacting protein 1 (PIK3IP1) is downregulated. The dysregulation of the Phosphoinositide-3-kinase (PI3K) pathway is associated with autoimmune disease [41]. Ovulation processes after luteinizing hormone stimulus involves steroids, prostaglandins, chemokines, and cytokines, which are also mediators of inflammatory processes. These mediators activate resident immune cells within the ovary [27]. Granulosa and theca cells of the follicle collaborate with immune cells to produce mediators of ovulation, many of which are also common to inflammatory responses.

A differential underexpressed gene in the OR line compared with the control line observed was Ubiquitin carboxyl-terminal esterase L1 (UCHL1). Its relevant functions are being anti-apoptotic [42,43], a tumor suppressor by inducing G0/G1cell cycle arrest [43], and enhancement of cell proliferation [44]. UCHL1 is highly and specifically expressed in mouse ova and is involved in the polyspermy block [45]. Moreover, UCHL1 is also involved in oocyte development and maturation [46,47]. All mentioned functions could play an important role to explain differences in prenatal survival between the selected and the control line. In our experiment, the proportion of females from the selected line with extremely high OR (more than 20 ova; i.e., twice the standard deviation over the mean) increased from 4% to 23% after ten generations of OR selection. Similar to superovulated females, which release oocytes that are less competent (reviewed by Krisher, 2004), females with extremely high ovulation rates from OR line could ovulate oocytes in an early stage of development, which may not be fertilized or may lead to poor-quality embryos that may die either before or after implantation.

Moreover, the angiogenesis process may be blocked by underexpression of regulator of G-protein signaling 22 (RGS22) [48] and coagulation factor II (thrombin) receptor (F2R) [49,50] in the OR line. The non-presence of ovarian angiogenic factors could produce (i.e., vascular epidermal growth factor (VEGF)) damage vascular density necessary during follicular development and the competence of the oocytes [51]. Angiogenesis increased vascular permeability, both vasodilation and vasoconstriction, and edema are essential features of ovulation.

Adiponectin (ADIPOQ) is an adipocyte-derived cytokine present in many reproductive tissues [52]. Their biological functions have expanded from insulin sensitization properties to new effects on inflammation and immunology [52]. Cytokines are inflammatory mediators that stimulate extensive remodeling of the extracellular matrix within the follicle [27]. Others roles are highlighted in fetal growth [53,54,55,56], in inhibitory effects on steroidogenesis [57,58], and progesterone secretion by the corpus luteum of the ovary [59]. Furthermore, higher levels of adiponectin are associated with better outcomes in assisted reproductive cycles [52] and success of embryo development [60] in fertile-cows [61]. In the present study the ADIPOQ gene was underexpressed, another author observed that the non-presence could be one of the main causes of infertility and associated with pregnancy-related disorders, including polycystic ovarian syndrome [56]. The last candidate, secreted frizzled-related protein 2 (SFRP2), family structurally related to Frizzled (Fz) proteins [62], is found downregulated in the OR line and could activate apoptosis [63] and disturbance in the development of embryogenesis [64]. Bone morphogenetic protein (BMP) [65], nuclear receptor subfamily 1 group H member 4 (NR1H4) [66] and carbonic anhydrase II (CA2) [67] could also contribute to the dysregulation of apoptosis. For this finding, the underexpression of these genes in the OR line vs. the control line could affect the correct development of ovarian tissue in the females belonging to the OR line. Specifically, CA2 was present in the endometrium of the non-pregnant rabbit in a low concentration, with a development of the endometrial activity declined [68].

## 5. Conclusions

The causes of prenatal mortality are a subject of great interest in productive species, such as the rabbit, and in assisted reproduction in humans. The results of this work have highlighted a set of genes that can help to understand better the mechanisms of oocyte maturation and ovulatory process and its relationship with the increase in prenatal mortality in rabbits and other species.

## Figures and Tables

**Table 1:** The most representative biological processes based on enrichment *p*-values in ovarian tissue.

Biological Processes	Number of Genes	Enrichment *p*-Value
Multicellular organismal development	55	1.81 × 10^−10^
Cell differentiation	43	8.80 × 10^−10^
Signal transduction	99	1.72 × 10^−09^
Synaptic transmission	29	8.25 × 10^−09^
Apoptotic process	40	3.18 × 10^−08^
Positive regulation of cell proliferation	29	3.50 × 10^−07^
Transmembrane transport	34	6.37 × 10^−07^
Positive regulation of transcription, DNA-templated	31	7.15 × 10^−07^
Positive regulation of I-kappaB kinase-NF-kappaB signaling	14	9.55 × 10^−07^

**Table 2:** Relevant cell process pathways based on *p*-values in ovarian tissue.

Cell Process Pathways	Number of Genes	Enrichment *p*-Value
Apoptosis	10	3.63 × 10^−03^
Melanogenesis	40	4.90 × 10^−03^
Translation Control	54	5.51 × 10^−03^
Actin Cytoskeleton Regulation	31	1.74 × 10^−02^
Thromboxane Receptor—“CREB signaling”	8	2.71 × 10^−02^
Chromosome Condensation	3	3.96 × 10^−02^
Lipoyl-protein complex biosynthesis II	2	4.17 × 10^−02^
Lipoyl-protein complex biosynthesis I	2	4.17 × 10^−02^
Cleavage of Lamina in Apoptosis	3	4.35 × 10^−02^
Histone Phosphorylation	9	4.43 × 10^−02^

**Table 3:** Nineteen significant differentially expressed genes involved in cell death in ovarian tissue.

Gene Symbol	Gene Name	*p*-Value	Fold Change
STC1	Stanniocalcin 1	1.11 × 10^−02^	1.90
IDO1	Indoleamine 2.3-dioxygenase 1	8.58 × 10^−03^	1.89
APOD	Apolipoprotein D	4.76 × 10^−02^	1.88
ENPP2	Ectonucleotide pyrophosphatase/phosphodiesterase 2	2.76 × 10^−02^	1.78
WEE2	WEE1 homolog 2 (*S. pombe*)	4.26 × 10^−02^	1.62
CDK1	Cyclin-dependent kinase 1	4.09 × 10^−02^	1.55
CBFB	Core-binding factor. beta subunit	4.20 × 10^−02^	1.53
CD38	CD38 molecule	3.75 × 10^−02^	1.52
RGS22	Regulator of G-protein signaling 22	2.69 × 10^−02^	−1.53
STK17A	Serine/threonine kinase 17a	1.77 × 10^−02^	−1.54
ADIPOQ	Adiponectin. C1Q and collagen domain containing	1.77 × 10^−02^	−1.57
CCL21	Chemokine (C-C motif) ligand 21	3.56 × 10^−02^	−1.64
UCHL1	Ubiquitin carboxyl-terminal esterase L1	2.84 × 10^−02^	−1.69
F2R	Coagulation factor II (thrombin) receptor	1.80 × 10^−02^	−1.70
PIK3IP1	Phosphoinositide-3-kinase interacting protein 1	4.65 × 10^−02^	−1.77
SFRP2	Secreted frizzled-related protein 2	2.20 × 10^−02^	−1.88
BMP5	Bone morphogenetic protein 5	3.72 × 10^−02^	−1.97
NR1H4	Nuclear receptor subfamily 1 group H member 4	1.31 × 10^−02^	−2.03
CA2	Carbonic anhydrase II	2.81 × 10^−02^	−2.75

## Supplementary Materials

The following are available online at https://www.mdpi.com/2076-2615/10/10/1924/s1, Table S1: 1357 differentially expressed genes between females from the line selected for ovulation rate during 10 generations (OR line) vs. the control line (C line).
