# Supplementary material for: Ovarian Transcriptomic Analysis Reveals Differential Expression Genes Associated with Cell Death Process after Selection for Ovulation Rate in Rabbits
Source: Animals (Basel). 2020 Oct 20;10(10):1924. doi: 10.3390/ani10101924 (PMC7593938; doi:10.3390/ani10101924)
Supplement: Supplementary file 1 [file animals-10-01924-s001.pdf]

Table S1. 1,357 differentially expressed genes between females from the line selected for ovulation rate during 10 generations (OR line) vs. the control line (C line).

Gene Symbol	*P*-value (OR line vs. C line)	Fold Change(OR line vs. C line)
C16H1orf131	1.39×10^{-02}	2.9356
STC1	1.11×10^{-02}	1.8976
IDO1	8.58×10^{-03}	1.89147
APOD	4.76×10^{-02}	1.87518
ENPP2	2.76×10^{-02}	1.78209
LOC100356950	3.88×10^{-02}	1.65936
WEE2	4.26×10^{-02}	1.61615
LOC100337961	2.34×10^{-02}	1.59155
CDK1	4.09×10^{-02}	1.55307
CBFB	4.20×10^{-02}	1.53402
ZNF367	2.48×10^{-02}	1.53233
CD38	3.75×10^{-02}	1.51654
LOC100339957	9.30×10^{-03}	1.51507
GNA14	4.37×10^{-03}	1.50342
ZP2	2.72×10^{-02}	1.49726
TMEM144	1.17×10^{-03}	1.4924
LOC103347295	1.19×10^{-02}	1.46929
CD40	3.09×10^{-02}	1.45909
LOC100342466	2.01×10^{-02}	1.44664
LOC100338257	9.98×10^{-03}	1.44631
FAM186A	7.87×10^{-03}	1.44506
DDX11	4.04×10^{-02}	1.44149
FAM72A	1.39×10^{-02}	1.43634
EOGT	2.98×10^{-02}	1.43554
PLEKHB1	4.36×10^{-03}	1.43432
NOS2	2.41×10^{-03}	1.43336
LOC103346704	1.36×10^{-02}	1.42876
LOC100338298	4.88×10^{-02}	1.42639
BPIFB6	3.45×10^{-02}	1.42558
LOC100352246	2.30×10^{-03}	1.41818
RTKN2	3.39×10^{-02}	1.41727
ORYCUNV1R1619	4.47×10^{-02}	1.41387
C16H1orf198	8.51×10^{-03}	1.40819
LOC100351812	3.14×10^{-02}	1.39873
ITM2C	1.04×10^{-02}	1.39832
LOC100356607	4.47×10^{-02}	1.39817
PRRT3	2.58×10^{-02}	1.3963
LOC100359295	2.21×10^{-02}	1.3954
PTDSS1	2.43×10^{-02}	1.39462
B3GALT5	3.45×10^{-02}	1.386
PEG10	3.17×10^{-02}	1.37964
LOC100340211	1.62×10^{-02}	1.3767
EIF5A2	3.40×10^{-02}	1.37549
EHF	7.57×10^{-03}	1.3744
DUT	2.70×10^{-02}	1.37312
LYVE1	3.61×10^{-02}	1.36957
SLCO3A1	4.95×10^{-02}	1.36857

Table S1. 1,357 differentially expressed genes between females from the line selected for ovulation rate during 10 generations (OR line) vs. the control line (C line).

TSTD2	1.06×10^{-02}	1.36752
MRPS33	2.14×10^{-02}	1.36436
LOC100338297	3.98×10^{-03}	1.36413
FHL2	2.57×10^{-02}	1.36317
PDZD4	1.68×10^{-02}	1.36122
EGFL8	1.59×10^{-02}	1.36001
HNRNPA0	9.48×10^{-03}	1.35742
LOC100340744	3.45×10^{-03}	1.353
LOC103345660	9.93×10^{-03}	1.35175
FAM207A	1.35×10^{-02}	1.35094
SPINK8	8.05×10^{-03}	1.35031
SDK1	4.52×10^{-02}	1.34975
GCSH	1.55×10^{-02}	1.34948
PDAP1	6.85×10^{-03}	1.34825
CRYBB2	1.92×10^{-03}	1.34543
GNG8	1.71×10^{-02}	1.34351
TMEM9B	2.94×10^{-02}	1.3429
LOC100342225	3.24×10^{-02}	1.33721
LOC103345170	6.94×10^{-03}	1.33535
CERCAM	2.50×10^{-04}	1.33295
SLC11A1	1.14×10^{-02}	1.3279
PPFIA4	4.92×10^{-02}	1.32584
LOC103346094	4.62×10^{-02}	1.32542
LOC100350060	1.62×10^{-02}	1.32424
AHNAK2	1.95×10^{-02}	1.32351
WDR31	1.01×10^{-02}	1.31955
ABCA8	2.28×10^{-02}	1.31931
CLDN6	2.50×10^{-02}	1.31632
LHX1	2.69×10^{-02}	1.31491
SSTR1	1.78×10^{-02}	1.31179
FAM167A	9.34×10^{-03}	1.31161
OSGEP	7.25×10^{-03}	1.31119
LOC103347875	1.89×10^{-02}	1.30967
ADAM6A	2.56×10^{-02}	1.30481
LOC100342855	2.06×10^{-02}	1.30224
LOC100346738	1.49×10^{-02}	1.29982
TIMM8B	1.08×10^{-02}	1.29978
TMEM213	9.24×10^{-03}	1.29815
PMCH	1.39×10^{-03}	1.29747
LOC100344262	4.61×10^{-02}	1.29564
LOC100342724	1.31×10^{-02}	1.29548
LOC100357544	2.42×10^{-02}	1.29459
FAM92B	3.93×10^{-02}	1.29385
RPL9	4.62×10^{-03}	1.29375
NKAIN1	3.54×10^{-02}	1.29365
LOC100340362	3.50×10^{-02}	1.29357
SLC25A14	1.17×10^{-02}	1.29322
LHX5	4.65×10^{-02}	1.29292

Table S1. 1,357 differentially expressed genes between females from the line selected for ovulation rate during 10 generations (OR line) vs. the control line (C line).

LOC103350632	2.00×10^{-02}	1.2918
CDH24	4.97×10^{-02}	1.29123
GPR17	1.62×10^{-02}	1.2894
NOL4L	4.87×10^{-03}	1.28798
LOC103346832	1.40×10^{-02}	1.28696
LOC103348194	1.99×10^{-02}	1.28646
LOC100351379	1.94×10^{-02}	1.28578
DYX1C1	4.12×10^{-02}	1.28358
C16H1orf131	3.10×10^{-02}	1.28274
CCNE2	2.46×10^{-02}	1.2801
FEN1	9.77×10^{-04}	1.28009
NCK2	2.25×10^{-03}	1.27771
AMIGO3	2.52×10^{-03}	1.27768
RBMXL2	3.66×10^{-02}	1.27488
LHB	4.93×10^{-02}	1.27452
LOC100353270	4.65×10^{-02}	1.27451
TTC39A	9.95×10^{-04}	1.27269
THRSP	4.71×10^{-02}	1.27254
C17H14orf119	4.06×10^{-03}	1.27064
FAM203A	2.03×10^{-02}	1.27009
LRRN4CL	4.25×10^{-02}	1.26963
ORYCUNV1R1640	2.58×10^{-02}	1.26948
TUBB3	2.68×10^{-02}	1.26759
SMIM4	7.14×10^{-03}	1.26728
UTP11L	2.13×10^{-02}	1.26637
CLPS	3.92×10^{-02}	1.26561
SLC16A5	3.13×10^{-02}	1.26493
LOC100339705	3.71×10^{-03}	1.26342
LOC100347015	7.82×10^{-03}	1.26261
LRRC59	1.44×10^{-02}	1.26154
CNTNAP1	4.40×10^{-02}	1.26099
LOC100356940	7.59×10^{-03}	1.26068
ARID3B	2.04×10^{-02}	1.25783
LOC103345020	1.21×10^{-02}	1.25452
CNNM1	2.10×10^{-02}	1.25382
WNT11	2.65×10^{-02}	1.25368
LOC100359181	5.07×10^{-03}	1.25299
ATP4B	3.75×10^{-02}	1.25284
LOC100347417	1.54×10^{-03}	1.25135
HINT2	1.11×10^{-02}	1.24974
PROM2	1.69×10^{-02}	1.24966
ADORA1	1.24×10^{-02}	1.24934
QTRTD1	3.73×10^{-02}	1.24688
KCNE1	3.79×10^{-02}	1.24633
LOC100339956	3.98×10^{-02}	1.24581
CAPN6	4.35×10^{-02}	1.24556
LOC100343356	4.13×10^{-02}	1.24473
CHRNA2	3.99×10^{-02}	1.24344

Table S1. 1,357 differentially expressed genes between females from the line selected for ovulation rate during 10 generations (OR line) vs. the control line (C line).

CREB3L1	4.10×10^{-03}	1.24307
KIAA0895L	4.29×10^{-02}	1.24292
RGS2	4.49×10^{-02}	1.24252
AQP4	4.49×10^{-02}	1.24236
LOC103351879	9.71×10^{-03}	1.24232
ALDH1A1	3.26×10^{-02}	1.24114
LOC100342497	2.76×10^{-02}	1.23999
PAAF1	1.75×10^{-02}	1.23927
LOC100340749	8.79×10^{-03}	1.2391
LOC103346356	3.53×10^{-02}	1.23807
LYPD6	4.31×10^{-02}	1.23806
NRIP2	2.88×10^{-02}	1.23804
APOLD1	1.10×10^{-02}	1.23738
LOC100341420	2.84×10^{-02}	1.23721
ANHX	1.11×10^{-02}	1.23714
PET117	3.19×10^{-02}	1.23708
CSF1	6.46×10^{-03}	1.23622
PAG1	4.52×10^{-02}	1.23583
LOC100338878	4.40×10^{-02}	1.23535
MAK16	3.65×10^{-02}	1.23458
LOC100348955	3.59×10^{-02}	1.23448
LOC100358202	3.43×10^{-02}	1.23386
EIF4B	2.22×10^{-03}	1.23286
RBFOX2	4.31×10^{-02}	1.23283
LOC100341365	3.04×10^{-02}	1.2325
FAM83D	1.49×10^{-03}	1.23088
MDFI	4.01×10^{-02}	1.22977
HSF2BP	3.91×10^{-02}	1.22954
FBXL18	3.31×10^{-02}	1.22907
ZNF513	3.69×10^{-02}	1.22834
NLRC3	2.11×10^{-02}	1.22758
TMEM201	2.28×10^{-02}	1.22704
NCF4	2.53×10^{-02}	1.22692
NTM	3.61×10^{-02}	1.22677
CCDC8	1.74×10^{-03}	1.22668
GPR179	5.00×10^{-02}	1.22611
TRIM72	4.87×10^{-02}	1.22606
NMB	4.00×10^{-03}	1.22526
INHBC	3.16×10^{-02}	1.22519
PAK7	4.08×10^{-02}	1.22487
CCNJL	3.88×10^{-02}	1.22386
ACBD4	1.20×10^{-02}	1.22376
PAX3	1.04×10^{-02}	1.22368
NFYC	3.59×10^{-02}	1.22247
FLYWCH2	7.80×10^{-03}	1.22184
LOC100345569	3.27×10^{-04}	1.22164
FAM209B	1.41×10^{-02}	1.22129
NDUFA8	3.41×10^{-03}	1.22105

Table S1. 1,357 differentially expressed genes between females from the line selected for ovulation rate during 10 generations (OR line) vs. the control line (C line).

RUFY4	3.10×10^{-02}	1.22056
NAGLU	4.25×10^{-02}	1.22017
TMEM189	1.96×10^{-02}	1.22001
HLF	5.67×10^{-04}	1.21991
FAM46B	3.70×10^{-02}	1.21892
SPEF1	3.33×10^{-02}	1.21862
TNFAIP3	1.93×10^{-03}	1.21772
CHRNA5	4.37×10^{-02}	1.21741
IMPA2	4.61×10^{-02}	1.21704
BPIFB2	9.27×10^{-03}	1.21666
ELF5	7.95×10^{-03}	1.21632
LSMEM1	5.99×10^{-03}	1.21553
A2ML1	1.34×10^{-02}	1.21442
LOC103345029	4.63×10^{-02}	1.21435
FLRT1	3.62×10^{-02}	1.21431
CCDC11	3.82×10^{-02}	1.21271
SREK1IP1	1.31×10^{-02}	1.21235
LOC100339919	4.02×10^{-02}	1.21209
LOC100351809	4.98×10^{-02}	1.21195
YBX3	3.11×10^{-02}	1.21172
JMJD7	2.47×10^{-02}	1.21162
TTC21A	3.46×10^{-02}	1.21087
ZSCAN21	2.33×10^{-02}	1.21042
ACTR5	1.56×10^{-02}	1.21021
FGF21	1.47×10^{-02}	1.20889
OMA1	3.06×10^{-02}	1.20877
SLC17A2	1.11×10^{-02}	1.2086
LOC100339901	2.79×10^{-02}	1.20784
ADCY8	9.67×10^{-03}	1.20744
PPP1R15B	2.46×10^{-02}	1.20733
CDC6	1.09×10^{-03}	1.20619
DLG5	9.22×10^{-03}	1.20618
LOC103345424	7.06×10^{-03}	1.20601
JADE2	3.96×10^{-02}	1.2055
ECE1	2.56×10^{-02}	1.20542
COPS7B	4.51×10^{-02}	1.20489
SRF	1.80×10^{-02}	1.20427
RASSF8	3.92×10^{-02}	1.20305
LOC100354063	2.46×10^{-02}	1.20295
COL27A1	2.34×10^{-02}	1.20249
LOC100352221	4.80×10^{-02}	1.20201
RXRB	1.91×10^{-02}	1.20094
LTB4R2	2.26×10^{-02}	1.20085
LOC103347032	3.57×10^{-03}	1.20035
CDH23	5.76×10^{-03}	1.19923
NFASC	3.47×10^{-02}	1.19885
GLP2R	4.42×10^{-02}	1.19828
ZNF521	3.59×10^{-02}	1.19624

Table S1. 1,357 differentially expressed genes between females from the line selected for ovulation rate during 10 generations (OR line) vs. the control line (C line).

LOC100354257	2.10×10^{-02}	1.1955
ZC3H8	1.21×10^{-02}	1.19531
MAP3K6	3.95×10^{-02}	1.19513
DDX27	3.10×10^{-02}	1.195
VSIG10	6.67×10^{-03}	1.195
MRC2	8.86×10^{-03}	1.1949
SIX4	3.89×10^{-02}	1.19447
AHSG	4.36×10^{-02}	1.19424
KLF15	4.75×10^{-02}	1.19383
INIP	3.47×10^{-02}	1.19366
GJC1	3.43×10^{-02}	1.19317
IGFN1	2.24×10^{-02}	1.19292
GPANK1	3.91×10^{-02}	1.19246
PITPNM3	1.24×10^{-02}	1.19237
CACNB3	3.93×10^{-02}	1.19215
PARK2	4.36×10^{-02}	1.19209
OVCH2	8.82×10^{-03}	1.19079
LOC100358738	4.68×10^{-02}	1.19029
BCL2L10	5.26×10^{-03}	1.18983
SCN3B	6.37×10^{-03}	1.18976
LOC100356855	3.15×10^{-02}	1.18946
SLC25A12	6.47×10^{-03}	1.18909
CASP16	3.75×10^{-02}	1.18879
NRG1	3.11×10^{-02}	1.18874
RELL2	6.36×10^{-03}	1.18831
LOC100337924	4.41×10^{-02}	1.18814
VPS37C	2.05×10^{-02}	1.18735
NYX	2.07×10^{-02}	1.18702
LOC100349954	3.98×10^{-02}	1.18659
LOC100351904	1.32×10^{-02}	1.18641
FAM183A	8.68×10^{-03}	1.18601
CTBP2	9.82×10^{-03}	1.18583
LOC100350386	3.17×10^{-02}	1.18579
ANKS6	4.19×10^{-02}	1.18578
SLC44A3	7.69×10^{-03}	1.18516
MKX	1.62×10^{-02}	1.18473
LOC103345397	4.41×10^{-02}	1.18456
TMEM14A	3.36×10^{-02}	1.18456
LOC103351165	4.73×10^{-02}	1.1844
MST1	1.13×10^{-02}	1.18394
GRXCR2	3.97×10^{-02}	1.18373
FOXN4	3.20×10^{-02}	1.18305
NLRP8	4.57×10^{-02}	1.18285
PAK4	3.27×10^{-02}	1.18276
P2RX3	1.78×10^{-02}	1.1825
RABEP2	1.46×10^{-02}	1.1822
NUFIP1	3.41×10^{-02}	1.182
CCDC13	2.86×10^{-02}	1.18177

Table S1. 1,357 differentially expressed genes between females from the line selected for ovulation rate during 10 generations (OR line) vs. the control line (C line).

GPR84	6.31×10^{-04}	1.18177
C13H1orf210	2.19×10^{-03}	1.18162
ADRA1B	4.96×10^{-02}	1.18039
UCK2	1.74×10^{-02}	1.17996
RCN2	2.51×10^{-02}	1.17957
CPN2	4.28×10^{-02}	1.17924
CHST10	1.08×10^{-02}	1.1789
NFKBIA	3.98×10^{-02}	1.17834
GLIS1	7.59×10^{-03}	1.17762
LOC100341759	4.78×10^{-02}	1.1773
IL31RA	3.23×10^{-02}	1.1765
PSORS1C2	1.34×10^{-02}	1.17633
LOC100358238	2.50×10^{-02}	1.1762
CDC25A	4.71×10^{-02}	1.17557
NUDT3	3.30×10^{-02}	1.17551
SSTR2	1.50×10^{-02}	1.1751
ROR2	1.26×10^{-02}	1.17485
MPC2	2.36×10^{-02}	1.17469
COL2A1	2.35×10^{-02}	1.17466
PDCD2	2.18×10^{-02}	1.1744
LOC100354647	8.93×10^{-03}	1.17342
PDIA5	4.07×10^{-02}	1.17309
TNFAIP8L3	2.76×10^{-02}	1.17296
LOC103350105	4.79×10^{-02}	1.1726
NRP2	1.91×10^{-02}	1.17218
FAM166B	3.10×10^{-02}	1.17159
WNT4	3.52×10^{-02}	1.17081
CHCHD3	3.88×10^{-02}	1.17057
IGSF9B	2.21×10^{-02}	1.16987
DTX4	7.02×10^{-03}	1.16979
ABLIM1	4.15×10^{-02}	1.16885
ZNF831	7.62×10^{-03}	1.16885
SLA2	2.18×10^{-02}	1.16877
AVP	1.97×10^{-02}	1.16842
C13H1orf56	3.67×10^{-04}	1.16837
REEP2	2.66×10^{-02}	1.16731
LOC100357720	2.79×10^{-02}	1.16723
SGCZ	4.71×10^{-02}	1.16712
BGN	1.86×10^{-02}	1.16709
CBX5	1.64×10^{-02}	1.16683
CCKBR	1.16×10^{-02}	1.16589
LOC100352315	4.31×10^{-02}	1.16531
CELF6	3.44×10^{-02}	1.16416
TMEM82	2.51×10^{-02}	1.16373
NUDT22	4.16×10^{-02}	1.16361
CLIC2	1.44×10^{-02}	1.16344
MUSK	4.42×10^{-02}	1.16339
SRCRB4D	2.06×10^{-02}	1.16334

Table S1. 1,357 differentially expressed genes between females from the line selected for ovulation rate during 10 generations (OR line) vs. the control line (C line).

PLXNA3	1.02×10^{-02}	1.1626
FAM160A1	3.50×10^{-02}	1.16249
ITIH4	3.56×10^{-02}	1.16147
ST3GAL4	3.92×10^{-02}	1.16101
CLK3	1.80×10^{-02}	1.16099
KISS1	3.05×10^{-03}	1.16064
MOCOS	1.40×10^{-02}	1.15926
MRPL15	7.31×10^{-03}	1.15854
LOC100339689	1.37×10^{-02}	1.15837
LOC100346078	2.94×10^{-02}	1.15836
LOC100345144	3.83×10^{-02}	1.15792
MFSD4	5.96×10^{-03}	1.15788
TMC3	2.53×10^{-02}	1.15748
SLC28A3	3.37×10^{-02}	1.15669
ACAP1	2.87×10^{-02}	1.15648
AIFM1	4.01×10^{-02}	1.15646
S100A3	1.14×10^{-02}	1.15641
MUC21	4.98×10^{-02}	1.15637
TIMELESS	1.95×10^{-02}	1.15628
HS6ST3	3.64×10^{-02}	1.15596
LOC100338620	4.85×10^{-02}	1.15586
PLAGL1	4.17×10^{-02}	1.15566
WARS2	2.59×10^{-02}	1.1554
LOC100350250	1.41×10^{-03}	1.155
LOC100354652	2.08×10^{-03}	1.153
PASK	1.75×10^{-03}	1.15294
IFT27	9.55×10^{-03}	1.1524
PDE8A	2.89×10^{-02}	1.15215
NLRX1	1.03×10^{-02}	1.15162
ITGBL1	3.85×10^{-02}	1.15092
PTH1R	2.36×10^{-02}	1.15083
LOC103347992	2.94×10^{-02}	1.15082
NQO1	2.52×10^{-02}	1.15002
BTBD17	3.16×10^{-02}	1.1497
DNAJC24	2.26×10^{-02}	1.14835
CASS4	1.77×10^{-02}	1.1481
LOC100355768	1.32×10^{-02}	1.14809
C13H1orf234	3.13×10^{-02}	1.14796
TESK1	3.77×10^{-02}	1.14689
HSD17B2	4.48×10^{-02}	1.14677
SPEG	1.41×10^{-02}	1.14656
ARTN	1.03×10^{-02}	1.14585
LOC100341024	4.19×10^{-02}	1.14562
LOC100338033	2.13×10^{-02}	1.14501
LOC100352044	3.37×10^{-02}	1.14365
PSRC1	2.80×10^{-02}	1.14339
CAMK2N2	4.60×10^{-02}	1.14313
SLC35B2	4.21×10^{-02}	1.14302

Table S1. 1,357 differentially expressed genes between females from the line selected for ovulation rate during 10 generations (OR line) vs. the control line (C line).

LOC100354171	2.75×10^{-02}	1.14289
PFDN2	3.65×10^{-02}	1.14274
CRTC2	2.47×10^{-02}	1.14106
PITPNA	1.32×10^{-02}	1.14064
ORMDL3	3.09×10^{-02}	1.13971
GJC2	3.63×10^{-02}	1.13947
SNRPA1	6.99×10^{-03}	1.1393
GNG2	3.57×10^{-02}	1.13908
MTPAP	1.77×10^{-02}	1.13904
ERICH3	6.98×10^{-03}	1.13792
TTLL9	1.09×10^{-02}	1.13774
LOC100345310	3.09×10^{-02}	1.13768
TMC1	2.34×10^{-02}	1.13752
AKAP8	1.80×10^{-02}	1.13744
NR2E1	1.16×10^{-04}	1.13668
PBX1	1.86×10^{-02}	1.13654
SLC27A3	1.17×10^{-02}	1.13593
NUP35	2.58×10^{-02}	1.13556
MALL	1.11×10^{-02}	1.13555
GABRD	2.97×10^{-02}	1.1352
TNFRSF13C	1.66×10^{-02}	1.13455
CASP7	7.57×10^{-04}	1.13364
SALL1	4.66×10^{-02}	1.13277
KIAA1045	2.42×10^{-03}	1.13223
LOC100344915	4.94×10^{-02}	1.13222
DNASE1L2	1.06×10^{-02}	1.13148
BRF2	3.06×10^{-02}	1.13096
KLHL22	9.19×10^{-03}	1.13091
SLMO1	1.22×10^{-02}	1.13057
LOC100357377	3.89×10^{-02}	1.13007
FOXO4	3.94×10^{-02}	1.12997
MLST8	3.26×10^{-02}	1.1294
LOC100358729	1.39×10^{-03}	1.12925
NEURL3	4.46×10^{-02}	1.12903
WDR4	1.63×10^{-02}	1.12883
LOC100348443	7.56×10^{-03}	1.12837
RBBP9	8.33×10^{-03}	1.12773
TEX101	2.21×10^{-02}	1.12763
LOC103345308	2.97×10^{-02}	1.12715
LOC100348347	1.16×10^{-02}	1.12629
SFXN5	1.29×10^{-04}	1.12626
SNX20	4.72×10^{-02}	1.12609
CD99L2	7.76×10^{-03}	1.12582
CHST3	2.27×10^{-02}	1.12564
F10	3.21×10^{-02}	1.1249
ARNTL2	3.97×10^{-02}	1.12453
LITAF	2.21×10^{-02}	1.12453
DEFB110	2.05×10^{-02}	1.12445

Table S1. 1,357 differentially expressed genes between females from the line selected for ovulation rate during 10 generations (OR line) vs. the control line (C line).

MYO16	3.05×10^{-02}	1.1243
LOC100340984	3.42×10^{-02}	1.12331
FAM136A	4.13×10^{-02}	1.12297
NOB1	3.17×10^{-02}	1.1228
PLA2G5	2.36×10^{-02}	1.12241
AMZ1	8.03×10^{-03}	1.12042
POLR1D	3.61×10^{-03}	1.11974
LOC100354908	1.78×10^{-02}	1.11932
TDRD6	3.39×10^{-02}	1.11854
MICALCL	2.03×10^{-02}	1.11852
KIF17	4.80×10^{-02}	1.11837
LAMB3	2.88×10^{-02}	1.11823
RILPL1	1.78×10^{-02}	1.118
RPS6KA2	3.01×10^{-02}	1.11777
CST7	4.23×10^{-02}	1.11729
KDM1B	4.79×10^{-02}	1.11687
CALM1	1.25×10^{-02}	1.11664
TBC1D14	9.11×10^{-03}	1.11598
LOC100355448	3.44×10^{-02}	1.11542
TMEM215	3.79×10^{-02}	1.11518
FAM118A	4.03×10^{-02}	1.11514
ANKRD34C	1.54×10^{-02}	1.11202
MPZL1	4.10×10^{-03}	1.11089
LOC100343292	2.20×10^{-02}	1.10835
SLCO5A1	1.20×10^{-03}	1.10799
GPR157	2.51×10^{-02}	1.10685
LOC100337818	3.45×10^{-02}	1.10674
FBXO4	4.57×10^{-02}	1.10666
KCNJ1	2.98×10^{-02}	1.10634
LATS2	1.68×10^{-03}	1.10632
MUC3A	4.22×10^{-02}	1.10512
UBE2D3	4.12×10^{-02}	1.10497
LEPROTL1	3.00×10^{-02}	1.10411
ZNF217	2.17×10^{-02}	1.10401
FCGBP	5.17×10^{-03}	1.10391
LOC100349629	4.03×10^{-02}	1.1033
ZSCAN25	2.86×10^{-02}	1.10306
EFCAB4B	3.06×10^{-02}	1.10227
PPIB	2.46×10^{-02}	1.10218
TNFRSF1A	7.08×10^{-03}	1.10205
LOC100339518	4.54×10^{-02}	1.10177
CNFN	3.85×10^{-03}	1.09998
CACNA1B	1.07×10^{-02}	1.09836
LEMD2	2.48×10^{-02}	1.09491
TSPAN7	3.76×10^{-02}	1.09464
CIITA	1.94×10^{-02}	1.09314
STRA6	3.01×10^{-02}	1.09178
TMEM212	3.14×10^{-02}	1.09165

Table S1. 1,357 differentially expressed genes between females from the line selected for ovulation rate during 10 generations (OR line) vs. the control line (C line).

ZNF317	3.96×10^{-02}	1.09149
FAM155A	3.05×10^{-02}	1.09108
VSTM2B	2.50×10^{-02}	1.08948
UNC45B	2.82×10^{-02}	1.08931
RNF220	4.87×10^{-02}	1.08835
TRPV6	4.11×10^{-02}	1.08785
PFN2	4.65×10^{-02}	1.08771
SLC26A3	4.08×10^{-02}	1.08745
PTGDR	4.61×10^{-02}	1.08703
PFDN4	2.51×10^{-02}	1.08673
PLET1	1.91×10^{-02}	1.0861
LLGL2	1.54×10^{-02}	1.08544
DNAH3	4.63×10^{-02}	1.08411
TMEM52	2.97×10^{-02}	1.08323
SPHK1	1.89×10^{-02}	1.0831
GRAMD3	2.74×10^{-02}	1.08184
FTSJ3	2.75×10^{-02}	1.08169
GOLGA3	1.46×10^{-02}	1.08162
CRK	2.19×10^{-02}	1.08135
KRT4	2.82×10^{-02}	1.07968
WDTC1	1.20×10^{-02}	1.07949
NCEH1	4.32×10^{-02}	1.07853
NAT1	4.97×10^{-02}	1.07788
LOC100342451	1.37×10^{-02}	1.0773
PLEKHH3	3.19×10^{-02}	1.0764
SNRNP70	2.96×10^{-02}	1.07567
LOC100341926	3.52×10^{-02}	1.07556
LOC100339519	1.54×10^{-02}	1.07426
IL5RA	1.99×10^{-02}	1.07379
LGALS2	2.74×10^{-02}	1.07187
RTP2	1.37×10^{-03}	1.07178
EVPL	9.04×10^{-03}	1.07031
LOC100348379	1.19×10^{-02}	1.06839
DCAF8	3.71×10^{-02}	1.06831
RHNO1	3.30×10^{-02}	1.06568
NF2	4.32×10^{-02}	1.0656
FAF1	4.19×10^{-02}	1.06535
MRPS5	2.68×10^{-02}	1.06444
COPB2	4.58×10^{-02}	1.06341
RARG	2.77×10^{-02}	1.06205
LOC100008880	4.52×10^{-02}	1.06017
SAR1A	1.72×10^{-02}	1.05775
TMEM39A	3.14×10^{-02}	1.05763
ERN2	3.05×10^{-02}	1.05753
LSG1	3.47×10^{-02}	1.05397
SEMA5B	4.25×10^{-02}	1.05201
ITGB7	2.42×10^{-02}	1.05044
RGS16	3.60×10^{-02}	1.04673

Table S1. 1,357 differentially expressed genes between females from the line selected for ovulation rate during 10 generations (OR line) vs. the control line (C line).

LOC100341932	4.18×10^{-03}	1.03678
STARD10	3.22×10^{-02}	-1.02199
GLOD4	4.49×10^{-02}	-1.02366
FAM168B	2.35×10^{-02}	-1.03088
THOC3	2.77×10^{-02}	-1.03645
INTS4	4.65×10^{-02}	-1.04236
LOC100340079	1.13×10^{-02}	-1.04433
ORMDL1	1.64×10^{-03}	-1.04921
ASAP3	1.21×10^{-02}	-1.04994
DLL3	4.68×10^{-02}	-1.05012
CD200R1L	2.76×10^{-02}	-1.05478
STAMBP	1.82×10^{-02}	-1.05644
PPP1R2	4.67×10^{-02}	-1.05659
BEND3	1.85×10^{-02}	-1.0571
LOC100344007	3.93×10^{-03}	-1.05733
LOC103348230	4.38×10^{-02}	-1.05925
WDFY1	2.02×10^{-02}	-1.05954
PRTFDC1	3.29×10^{-02}	-1.06007
CYP3A6	4.03×10^{-02}	-1.06108
USP12	2.26×10^{-02}	-1.06116
HSPA12B	1.99×10^{-02}	-1.06598
OLA1	9.51×10^{-03}	-1.06692
EQTN	4.78×10^{-03}	-1.06742
PSMC1	6.17×10^{-03}	-1.07048
TARS	3.84×10^{-02}	-1.07139
RPRD2	4.95×10^{-02}	-1.07308
CHD4	1.06×10^{-02}	-1.07449
C3H8orf46	4.37×10^{-02}	-1.07493
IFNAR1	2.42×10^{-02}	-1.0756
PCP2	2.39×10^{-02}	-1.07599
RAB14	9.95×10^{-03}	-1.07727
TTC28	3.38×10^{-02}	-1.07751
RBM28	3.38×10^{-02}	-1.07781
MORC2	3.62×10^{-02}	-1.07861
PARN	4.62×10^{-02}	-1.08075
PCSK2	1.12×10^{-02}	-1.08105
BRAF	1.55×10^{-02}	-1.08124
KCNH2	1.23×10^{-03}	-1.08129
MYADML2	4.54×10^{-02}	-1.08144
SMAP2	4.39×10^{-02}	-1.08219
GNGT1	1.64×10^{-02}	-1.08355
KANSL2	7.30×10^{-03}	-1.0842
ZDHHC6	4.15×10^{-02}	-1.08588
ERCC4	3.33×10^{-04}	-1.08725
SPRR3	3.72×10^{-02}	-1.08944
HSDL1	1.27×10^{-02}	-1.08955
CCDC68	4.10×10^{-02}	-1.0916
HSDL2	1.17×10^{-02}	-1.09245

Table S1. 1,357 differentially expressed genes between females from the line selected for ovulation rate during 10 generations (OR line) vs. the control line (C line).

TATDN1	1.30×10^{-02}	-1.09256
ZBTB2	4.91×10^{-02}	-1.0926
ZNRF2	1.32×10^{-02}	-1.09294
SPARC	1.72×10^{-03}	-1.09387
EMX1	1.30×10^{-02}	-1.09674
LOC100348628	3.86×10^{-02}	-1.09682
IMMP1L	4.51×10^{-02}	-1.09722
TMEM87A	4.04×10^{-02}	-1.09729
LOC100353687	1.62×10^{-02}	-1.09787
DTNB	2.39×10^{-02}	-1.09869
TICAM2	3.07×10^{-02}	-1.0991
HIF1AN	3.34×10^{-02}	-1.1009
CYFIP1	1.24×10^{-02}	-1.10116
LOC100356418	2.66×10^{-02}	-1.10145
CORT	3.77×10^{-02}	-1.10147
HECTD4	1.86×10^{-02}	-1.10364
DNM1L	4.12×10^{-02}	-1.10389
LOC100356606	3.60×10^{-02}	-1.10433
TVP23B	3.46×10^{-03}	-1.10513
CDKL5	4.79×10^{-02}	-1.10534
TERF2IP	4.20×10^{-02}	-1.10637
ATG2B	4.64×10^{-02}	-1.10896
KMO	3.32×10^{-02}	-1.10988
ZNHIT6	2.22×10^{-02}	-1.10999
SLC17A5	2.31×10^{-02}	-1.11088
NOVA2	2.30×10^{-02}	-1.11179
LARK	1.79×10^{-02}	-1.11202
DOCK4	1.36×10^{-02}	-1.11256
GRM7	3.35×10^{-03}	-1.11286
ASZ1	1.83×10^{-02}	-1.11347
LOC100351515	3.05×10^{-02}	-1.11455
DNAH11	3.30×10^{-02}	-1.11484
LOC103347152	4.87×10^{-02}	-1.11518
CAPN3	2.21×10^{-02}	-1.11642
SRPK2	4.58×10^{-02}	-1.11736
TTC8	1.57×10^{-02}	-1.1177
GPR89B	1.47×10^{-02}	-1.11823
KCTD3	3.33×10^{-02}	-1.11998
MGME1	3.06×10^{-02}	-1.12003
ARHGEF37	3.37×10^{-02}	-1.1202
NEBL	2.19×10^{-02}	-1.12025
EIF2S3	8.26×10^{-03}	-1.12167
LOC100357308	3.45×10^{-02}	-1.12181
SAT2	3.07×10^{-02}	-1.12276
LOC100348189	6.28×10^{-03}	-1.12379
FZD7	3.00×10^{-02}	-1.12444
PDCL3	7.00×10^{-03}	-1.12456
PCBP3	3.45×10^{-02}	-1.12473

Table S1. 1,357 differentially expressed genes between females from the line selected for ovulation rate during 10 generations (OR line) vs. the control line (C line).

CLHC1	3.00×10^{-02}	-1.1255
USP21	1.35×10^{-02}	-1.1256
NDFIP2	4.41×10^{-03}	-1.12581
ANKRD28	4.92×10^{-02}	-1.1265
KIF1B	7.56×10^{-03}	-1.12662
SHOC2	4.02×10^{-02}	-1.12866
BAHD1	3.19×10^{-02}	-1.12904
DLG3	4.74×10^{-02}	-1.12906
LOC103345933	3.36×10^{-02}	-1.12912
C21H12orf49	2.13×10^{-02}	-1.12915
MAPK6	3.50×10^{-02}	-1.12918
IRAK3	1.64×10^{-03}	-1.12934
RFX5	4.29×10^{-03}	-1.12945
DNMBP	3.96×10^{-02}	-1.13048
LOC100347861	2.39×10^{-02}	-1.13061
GLCCI1	2.08×10^{-02}	-1.1322
SH3YL1	4.09×10^{-02}	-1.13224
CCDC90B	1.08×10^{-02}	-1.13263
GPC3	1.90×10^{-03}	-1.13263
ADCY4	8.93×10^{-04}	-1.13279
LOC100355132	4.12×10^{-02}	-1.13286
APPL1	4.18×10^{-02}	-1.13343
AHNAK	2.17×10^{-03}	-1.13437
HELQ	7.22×10^{-03}	-1.13486
LMNTD1	1.41×10^{-02}	-1.13568
SLC6A6	3.85×10^{-02}	-1.13608
NDUFAF5	3.42×10^{-02}	-1.13843
S1PR1	1.75×10^{-03}	-1.13962
SYNDIG1	1.28×10^{-02}	-1.14003
AR	3.78×10^{-02}	-1.14027
EIF2S2	3.30×10^{-02}	-1.14038
FUT10	1.56×10^{-02}	-1.14107
TXNL1	2.11×10^{-02}	-1.14134
RYR2	3.82×10^{-02}	-1.14154
CRYZL1	3.81×10^{-02}	-1.14227
C2H2orf43	1.88×10^{-03}	-1.14299
FRMD6	4.62×10^{-02}	-1.14314
SHQ1	8.59×10^{-03}	-1.14339
IFT81	3.07×10^{-02}	-1.14365
TYW3	2.09×10^{-02}	-1.1444
FKBP9	2.77×10^{-02}	-1.14523
TMED5	2.28×10^{-02}	-1.14659
DIO1	4.05×10^{-02}	-1.14719
GPR128	3.71×10^{-02}	-1.14741
DNAJC5B	4.23×10^{-02}	-1.14819
VEZT	4.24×10^{-02}	-1.14991
AOX1	2.86×10^{-03}	-1.1501
LOC103348208	3.76×10^{-02}	-1.15071

Table S1. 1,357 differentially expressed genes between females from the line selected for ovulation rate during 10 generations (OR line) vs. the control line (C line).

SPRYD7	3.00×10^{-02}	-1.15105
CNGB3	1.10×10^{-02}	-1.15218
CCDC37	7.71×10^{-04}	-1.15225
FUK	1.14×10^{-02}	-1.15234
LIN7A	4.24×10^{-02}	-1.15254
PHACTR4	1.33×10^{-02}	-1.15265
LYPLAL1	3.93×10^{-02}	-1.15295
NHLRC3	4.01×10^{-02}	-1.15355
SORBS2	1.55×10^{-02}	-1.15452
GIF	1.83×10^{-02}	-1.15502
ADAM10	4.27×10^{-02}	-1.15526
LOC100350209	3.11×10^{-02}	-1.15579
LOC100353262	4.58×10^{-02}	-1.1561
MRRF	3.59×10^{-03}	-1.15651
CNNM3	2.67×10^{-02}	-1.15675
PRDX3	2.77×10^{-02}	-1.15718
RNF138	4.03×10^{-02}	-1.15793
C1H9orf84	4.89×10^{-03}	-1.15813
PIK3CB	4.85×10^{-02}	-1.1582
KCNF1	4.23×10^{-02}	-1.15849
ZNF277	4.41×10^{-02}	-1.15984
ANKFY1	3.40×10^{-02}	-1.16021
GABRA4	3.26×10^{-02}	-1.16053
MCM9	9.55×10^{-03}	-1.16086
SPPL2C	4.78×10^{-02}	-1.16137
LOC103347338	5.67×10^{-03}	-1.16161
LOC100349830	3.75×10^{-02}	-1.16289
DDHD1	2.54×10^{-02}	-1.16343
INSC	2.03×10^{-02}	-1.16349
KIAA2026	3.03×10^{-02}	-1.16368
KIT	3.92×10^{-02}	-1.16398
WDR20	1.80×10^{-02}	-1.16467
LOC103351146	3.73×10^{-02}	-1.16562
RHBDD1	4.75×10^{-02}	-1.16563
ISCA2	2.24×10^{-02}	-1.16573
GFOD1	4.58×10^{-02}	-1.16618
LOC100350678	3.98×10^{-02}	-1.1669
LOC100341357	3.43×10^{-02}	-1.16737
DOCK1	4.47×10^{-02}	-1.16742
MAP1B	5.62×10^{-03}	-1.1677
TTLL7	3.11×10^{-02}	-1.16845
VPS13D	2.34×10^{-02}	-1.16899
RRNAD1	4.41×10^{-02}	-1.16981
CSMD2	2.38×10^{-03}	-1.17057
NDNL2	4.90×10^{-02}	-1.1706
SAMD8	3.19×10^{-02}	-1.17073
RGL2	6.31×10^{-03}	-1.17081
TRAPPC8	3.59×10^{-02}	-1.17106

Table S1. 1,357 differentially expressed genes between females from the line selected for ovulation rate during 10 generations (OR line) vs. the control line (C line).

ERI3	1.06×10^{-02}	-1.17216
SERINC1	2.38×10^{-02}	-1.17216
KLHL32	4.77×10^{-02}	-1.17252
EBF1	3.11×10^{-02}	-1.17262
SLC40A1	4.87×10^{-02}	-1.17432
ERAL1	8.60×10^{-04}	-1.17464
GPR63	1.72×10^{-02}	-1.17572
EFR3A	1.28×10^{-02}	-1.1761
MLH1	4.44×10^{-02}	-1.17629
EAPP	6.09×10^{-03}	-1.17753
PLEKHH1	4.67×10^{-02}	-1.17882
XPO4	2.67×10^{-02}	-1.17928
SYT11	3.55×10^{-02}	-1.18158
UBQLN3	1.48×10^{-02}	-1.18173
ISPD	7.52×10^{-03}	-1.18189
KLHL12	3.02×10^{-03}	-1.1822
ONECUT1	2.06×10^{-02}	-1.18242
KCTD15	3.13×10^{-03}	-1.18384
ZFHX3	3.67×10^{-02}	-1.18396
FAXDC2	3.29×10^{-02}	-1.18454
ABCC9	3.67×10^{-02}	-1.18513
ATP6V1C1	3.17×10^{-02}	-1.1854
LOC103350887	6.90×10^{-03}	-1.18572
MRAS	4.61×10^{-02}	-1.18615
C4H12orf55	3.59×10^{-02}	-1.1875
SNX19	1.60×10^{-02}	-1.1876
LOC100353656	4.92×10^{-03}	-1.18788
ECM1	2.17×10^{-02}	-1.18798
MORN4	4.07×10^{-02}	-1.18925
SLC26A7	4.21×10^{-02}	-1.18954
LOC103345097	2.16×10^{-02}	-1.19019
ARL4C	3.59×10^{-02}	-1.19021
P2RY14	3.52×10^{-02}	-1.19134
CNDP1	7.72×10^{-03}	-1.19157
LOC100341014	9.32×10^{-03}	-1.19312
SERINC3	2.53×10^{-02}	-1.19436
PNPT1	9.41×10^{-04}	-1.19437
LOC103348008	3.91×10^{-02}	-1.19691
PDE10A	1.08×10^{-02}	-1.19729
CCL14	2.51×10^{-03}	-1.19746
LOC100347831	4.69×10^{-02}	-1.19868
TAMM41	2.72×10^{-02}	-1.19876
UTP18	1.51×10^{-02}	-1.19954
PCMTD2	4.89×10^{-02}	-1.19967
GSTCD	1.31×10^{-02}	-1.20007
KCND3	1.52×10^{-02}	-1.20111
LOC100354366	1.60×10^{-02}	-1.20144
MCAM	3.73×10^{-02}	-1.20252

Table S1. 1,357 differentially expressed genes between females from the line selected for ovulation rate during 10 generations (OR line) vs. the control line (C line).

LOC100338177	1.37×10^{-02}	-1.20258
FRRS1L	2.21×10^{-02}	-1.20314
AMER3	1.70×10^{-02}	-1.20385
ZNF148	3.17×10^{-02}	-1.2053
PTPRG	2.59×10^{-03}	-1.20566
DYSF	6.88×10^{-03}	-1.20612
MIPEP	1.36×10^{-03}	-1.20658
LOC100346708	3.11×10^{-02}	-1.20988
TMEM87B	3.60×10^{-02}	-1.21088
AZIN1	3.38×10^{-02}	-1.2111
MICU3	2.48×10^{-02}	-1.21358
LOC100355734	1.00×10^{-02}	-1.21371
LOC100356378	4.37×10^{-02}	-1.21458
CCZ1	2.40×10^{-02}	-1.21469
UTRN	2.71×10^{-02}	-1.21536
GBE1	4.24×10^{-02}	-1.21606
PDE4A	3.83×10^{-03}	-1.21681
ARF5	3.09×10^{-02}	-1.2176
RELN	4.34×10^{-03}	-1.21782
TEFM	1.68×10^{-02}	-1.21831
MTMR12	3.00×10^{-02}	-1.21904
PIGC	1.37×10^{-02}	-1.2197
CPNE8	1.17×10^{-02}	-1.22099
KIAA0556	1.05×10^{-03}	-1.22107
TBC1D32	3.96×10^{-02}	-1.22252
PDZD2	3.02×10^{-02}	-1.22267
UHRF1BP1L	3.78×10^{-03}	-1.2238
HMCN1	1.49×10^{-02}	-1.22442
LBH	3.46×10^{-02}	-1.22581
TTC39B	4.85×10^{-02}	-1.22672
GPCPD1	2.50×10^{-02}	-1.22686
TTC12	1.94×10^{-02}	-1.22996
TMEM242	4.34×10^{-02}	-1.23047
EXOC1	2.06×10^{-02}	-1.23048
DYNC2LI1	1.62×10^{-02}	-1.23061
GID4	4.55×10^{-02}	-1.23104
SLC9A7	1.22×10^{-02}	-1.2316
PPHLN1	4.33×10^{-02}	-1.23244
XPC	4.39×10^{-02}	-1.23386
LOC100340438	3.02×10^{-02}	-1.23416
SPEF2	1.59×10^{-02}	-1.23423
EDNRA	4.16×10^{-02}	-1.23439
LOC100346342	4.65×10^{-02}	-1.23558
MAP2	4.15×10^{-02}	-1.23681
PGPEP1	3.00×10^{-02}	-1.23835
SHROOM3	3.88×10^{-02}	-1.23866
UBOX5	3.08×10^{-02}	-1.2395
SIK3	4.61×10^{-02}	-1.24215

Table S1. 1,357 differentially expressed genes between females from the line selected for ovulation rate during 10 generations (OR line) vs. the control line (C line).

LOC100356501	9.36×10^{-03}	-1.24381
PSMD5	4.69×10^{-02}	-1.24599
LOC100343441	3.86×10^{-02}	-1.24823
LOC103351165	3.46×10^{-02}	-1.25192
SLC35F5	1.01×10^{-02}	-1.25193
EPM2A	4.80×10^{-02}	-1.25282
ORYCUNV1R1512	2.53×10^{-02}	-1.25401
ABAT	4.33×10^{-02}	-1.25642
SPDEF	1.80×10^{-03}	-1.25797
PRKCE	1.43×10^{-02}	-1.25799
B4GALT6	3.55×10^{-02}	-1.2584
TMEM161B	9.12×10^{-03}	-1.25931
LOC103346243	2.37×10^{-02}	-1.25943
FUCA1	2.41×10^{-03}	-1.2603
FAM210A	2.27×10^{-02}	-1.26139
TST	4.76×10^{-02}	-1.26142
DGKB	4.85×10^{-02}	-1.26203
C18H10orf107	4.62×10^{-02}	-1.26291
TP53INP1	4.30×10^{-02}	-1.26592
GALNTL6	6.68×10^{-03}	-1.26605
SNTB1	2.82×10^{-02}	-1.26635
SLC38A6	1.48×10^{-03}	-1.26694
C2H4orf47	1.63×10^{-02}	-1.27134
ERI2	4.02×10^{-02}	-1.27243
DMD	4.55×10^{-02}	-1.27348
PIBF1	3.56×10^{-02}	-1.27362
KIAA1107	6.60×10^{-03}	-1.27581
TMEM66	1.79×10^{-02}	-1.27674
EDNRB	2.75×10^{-02}	-1.27706
LRRN3	1.31×10^{-02}	-1.27897
SCAMP2	3.26×10^{-02}	-1.27949
WDR72	2.08×10^{-02}	-1.28067
LOC100341851	2.05×10^{-02}	-1.2814
SSFA2	2.97×10^{-02}	-1.28327
RUNX2	1.14×10^{-02}	-1.28597
HMGCLL1	1.20×10^{-02}	-1.29004
ST8SIA4	1.14×10^{-02}	-1.29038
UVRAG	3.32×10^{-02}	-1.29449
CD180	3.00×10^{-02}	-1.29504
NSUN6	3.86×10^{-02}	-1.29714
CRIPT	3.51×10^{-02}	-1.30716
FCF1	1.09×10^{-02}	-1.30904
C11H5orf34	6.35×10^{-03}	-1.30927
HEXB	3.84×10^{-02}	-1.31152
MAOB	3.24×10^{-02}	-1.31287
LOC100343468	3.85×10^{-02}	-1.31297
PLOD1	3.74×10^{-02}	-1.32016
PKHD1L1	2.91×10^{-02}	-1.3221

Table S1. 1,357 differentially expressed genes between females from the line selected for ovulation rate during 10 generations (OR line) vs. the control line (C line).

IDI1	7.65×10^{-04}	-1.32397
SLC46A2	7.46×10^{-03}	-1.3254
AP4E1	1.03×10^{-02}	-1.32999
TAX1BP1	9.32×10^{-03}	-1.33564
P2RY12	4.07×10^{-02}	-1.33623
CHRM3	2.95×10^{-02}	-1.33852
LCAT	1.06×10^{-02}	-1.3417
FAM159B	4.60×10^{-04}	-1.34264
CLDN22	1.14×10^{-02}	-1.34589
CALCRL	4.85×10^{-02}	-1.34857
FAM126A	8.61×10^{-03}	-1.34899
IDE	2.90×10^{-02}	-1.35132
MAST4	3.21×10^{-03}	-1.35221
TXNRD3	2.55×10^{-02}	-1.35333
LOC103352412	1.30×10^{-02}	-1.35987
APH1B	2.09×10^{-02}	-1.36084
RNPEP	2.36×10^{-02}	-1.36163
BRK1	2.47×10^{-02}	-1.36202
ABHD3	1.43×10^{-02}	-1.36703
TPK1	1.22×10^{-02}	-1.36776
NUDT12	2.65×10^{-02}	-1.36788
TACC2	1.36×10^{-02}	-1.36966
GABRG1	4.37×10^{-02}	-1.36984
ADAMTSL1	4.37×10^{-02}	-1.37118
ALDH1L1	3.41×10^{-03}	-1.3715
OGFOD1	2.69×10^{-02}	-1.37707
GFRA1	1.51×10^{-02}	-1.38423
CERS3	1.11×10^{-02}	-1.38581
FREM1	3.13×10^{-02}	-1.38636
EGF	4.93×10^{-02}	-1.39346
NMU	4.62×10^{-02}	-1.40539
QPCT	8.67×10^{-03}	-1.40563
RASL11A	4.56×10^{-02}	-1.42561
LOC103346593	3.59×10^{-02}	-1.4319
LOC100349989	4.68×10^{-02}	-1.43229
PLIN2	6.07×10^{-03}	-1.44699
NDUFAF2	3.51×10^{-02}	-1.47811
HERC4	4.23×10^{-02}	-1.48372
LOC100338666	2.19×10^{-02}	-1.49967
SLC51A	2.69×10^{-02}	-1.50409
CNTN3	2.93×10^{-02}	-1.50717
SYT12	2.61×10^{-02}	-1.51914
RGS22	2.69×10^{-02}	-1.52624
STK17A	1.77×10^{-02}	-1.54009
NR6A1	2.05×10^{-02}	-1.54462
ADIPOQ	1.77×10^{-02}	-1.565
CCL21	3.56×10^{-02}	-1.63809
ACSL4	2.98×10^{-02}	-1.65853

Table S1. 1,357 differentially expressed genes between females from the line selected for ovulation rate during 10 generations (OR line) vs. the control line (C line).

UCHL1	2.84×10^{-02}	-1.69432
F2R	1.80×10^{-02}	-1.70231
PIK3IP1	4.65×10^{-02}	-1.77359
FABP2	1.33×10^{-02}	-1.83137
SFRP2	2.20×10^{-02}	-1.88418
BMP5	3.72×10^{-02}	-1.96703
NR1H4	1.31×10^{-02}	-2.03068
PLA2G2D	2.76×10^{-03}	-2.2881
PTGFR	1.51×10^{-02}	-2.64957
CA2	2.81×10^{-02}	-2.75072
